# Best Practice Guideline Development Methods: An Integrated Approach Based on the Registered Nurses' Association of Ontario's Methods

**DOI:** 10.1002/gin2.70052

**Published:** 2025-11-28

**Authors:** Lyndsay Howitt, Amy Burt, Nafsin Nizum, Christine Buchanan, Michelle Rey, Doris Grinspun

**Affiliations:** ^1^ Registered Nurses' Association of Ontario North York Ontario Canada

**Keywords:** best practice guideline, clinical practice guideline, evidence‐based nursing, evidence‐based practice, guideline development, systematic review

## Abstract

Since 1999, the Registered Nurses' Association of Ontario (RNAO) has developed best practice guidelines (BPG) to promote consistency and quality of evidence‐based care and improve patient, organization and health system outcomes. A distinguishing feature of RNAO's BPG development portfolio is its integration within RNAO's three‐pillar BPG programme. This programme supports health service and academic organizations, Best Practice Spotlight Organizations® (BPSO®), to systematically implement, monitor and evaluate BPGs. Through formal partnerships with these organizations, the BPSO network extends the reach of the BPG programme and provides RNAO with direct feedback from end users, strengthening the relevance of future guideline editions. This article describes RNAO's integrated BPG development methods, outlining how evidence‐based recommendations are made to support organizations implementing BPGs. Following extensive pre‐development work to shape the purpose and scope of each guideline, an interprofessional expert panel that includes people with lived experience is appointed. Using the Grading of Recommendations, Assessment, Development and Evaluation methods, the expert panel prioritizes research questions and systematic reviews are conducted to determine strong or conditional recommendations. Good practice statements are also included and resources to support guideline implementation and evaluation are formulated including fact sheets, RNAO Clinical Pathways™ and evaluation measures. Draft guidelines undergo external review to ensure relevance and usability. To best serve the needs of organizations implementing BPGs, ongoing efforts are being made to keep RNAO's methods up‐to‐date and incorporate evaluation data from implementing organizations into the guideline development process.

## Summary

- This article describes the Registered Nurses' Association of Ontario's (RNAO) integrated approach to best practice guideline (BPG) development, which follows a seven‐step process grounded in internationally recognized standards and is designed to support organizations implementing guidelines.
- A distinguishing feature of RNAO's BPG programme is its integration within a three‐pillar model: guideline development, guideline dissemination, implementation and sustainability and guideline monitoring and evaluation.
- RNAO partners with Best Practice Spotlight Organizations® to support systematic BPG implementation and evaluation of outcomes.
- The integrated model allows evidence to be translated directly into practice, while insights and evaluation data from implementing organizations have the potential to inform future guideline development.
- While guidelines aim to improve healthcare quality and reduce variation in care, specific strategies are needed to support their uptake. RNAO's model is designed to address this gap by enhancing applicability and providing methods to evaluate the impact of guideline implementation.

## 1 Introduction

The Registered Nurses' Association of Ontario (RNAO) is the professional association representing registered nurses, nurse practitioners and nursing students in Ontario, Canada. Since 1999, RNAO's Best Practice Guideline (BPG) programme has received funding from the Government of Ontario to advance evidence‐based practice through guideline development, implementation and evaluation [1].

RNAO develops BPGs on clinical, healthy work environment, health equity and health system topics. Examples include guidelines on the management of diabetic foot ulcers, [2] prevention of violence against health workers, [3] and 2SLGBTQI+ health equity [4]. To ensure rigour and transparency, BPGs are developed using the Grading of Recommendations Assessment, Development and Evaluation (GRADE) approach [5]. The development panel is comprised of experts including interprofessional team members and people with lived experience (PWLE) such as patients and caregivers. Each guideline includes recommendations for nurses and the interprofessional team, educators and policymakers, to promote consistency in clinical care and education, with the goal of improving health outcomes for people, communities and the health system [1].

Following the principle that evidence‐based knowledge should be universally accessible, RNAO's BPG are freely available online and used around the globe [1]. Unlike organizations who focus primarily on guideline development, RNAO places equal emphasis on developing guidelines, supporting their implementation into practice and equipping organizations to evaluate guideline implementation to understand their impact. The guideline development process is led by Master and PhD prepared registered nurses, guideline development methodologists (GDMs) who oversee each guideline from conceptualization to publication. In addition, nurses specializing in knowledge translation serve as implementation coaches, supporting Best Practice Spotlight Organizations® (BPSO®) to implement and evaluate guidelines in practice. BPSOs are health service or academic organizations that have formally partnered with RNAO to foster evidence‐based practice cultures through the systematic implementation and outcome evaluation of RNAO's BPGs [1].

**Figure 1:**
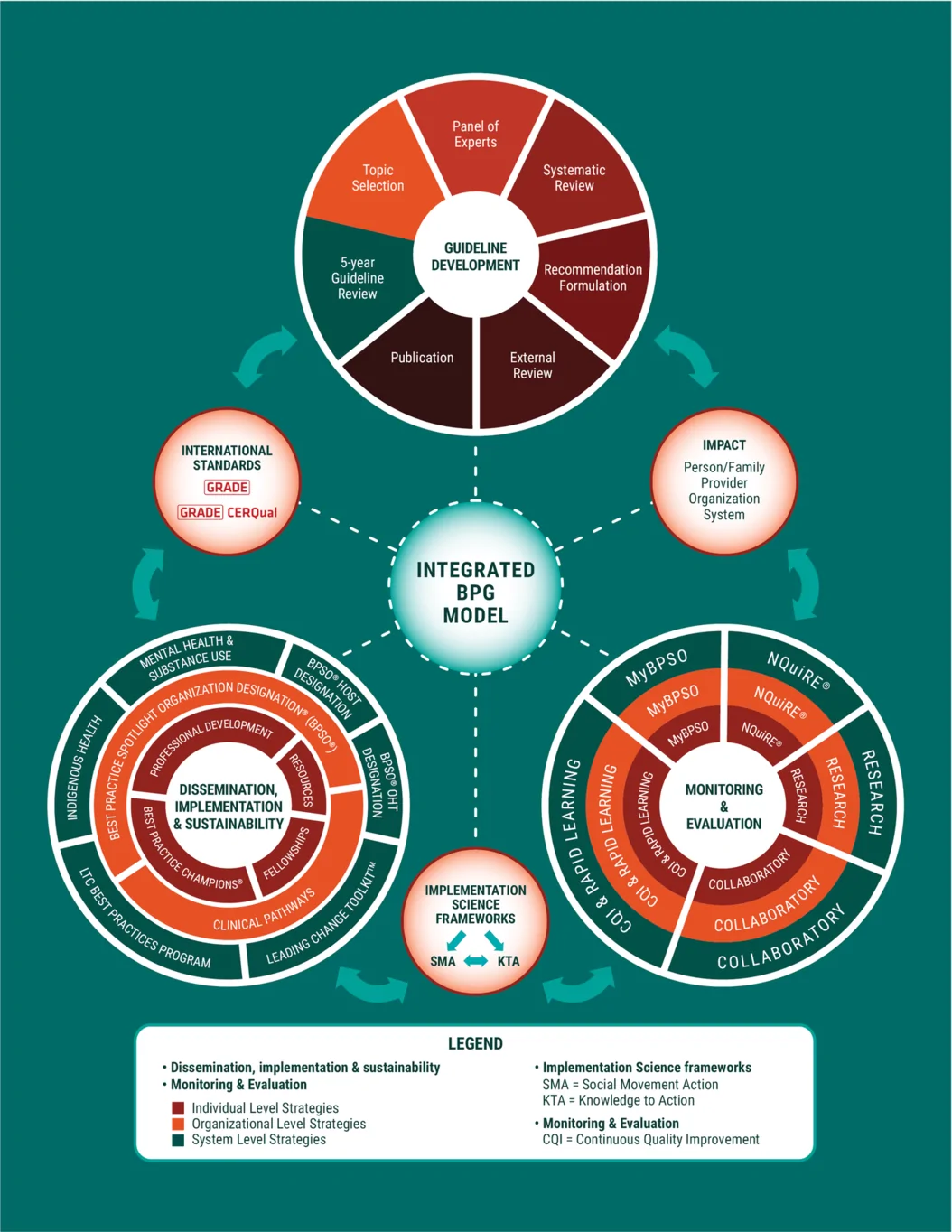
RNAO's integrated BPG model.

Figure 1 outlines RNAO's integrated BPG model that includes three pillars, equal in size: (1) guideline development; (2) guideline dissemination, implementation and sustainability and (3) guideline monitoring and evaluation. The guideline development pillar contains seven consecutive steps, while the implementation and evaluation pillars contain three concentric rings representing individual, organizational and health system level strategies. The integrated nature of the model facilitates a feedback loop: evidence is translated directly into practice, and the experiences of organizations implementing guidelines, and the evaluation data derived from BPG implementation, are fed back into the guideline development process. This integrated approach enhances the relevance and utility of the next edition BPGs for end users. The objective of this manuscript is to describe RNAO's integrated BPG development methods which serve to support organizations implementing BPGs.

## 2 Methods

To meet international standards for guideline development and reporting, RNAO has integrated the criteria outlined in the Appraisal of Guidelines for Research and Evaluation (AGREE II) instrument and the Reporting Items for practice Guidelines in HealThcare (RIGHT) statement into its internal guideline development handbook [6, 7]. As AGREE III is currently under development, RNAO will conduct a comprehensive review upon its publication and update internal procedures to ensure continued alignment with evolving standards [8].

**Figure 2:**
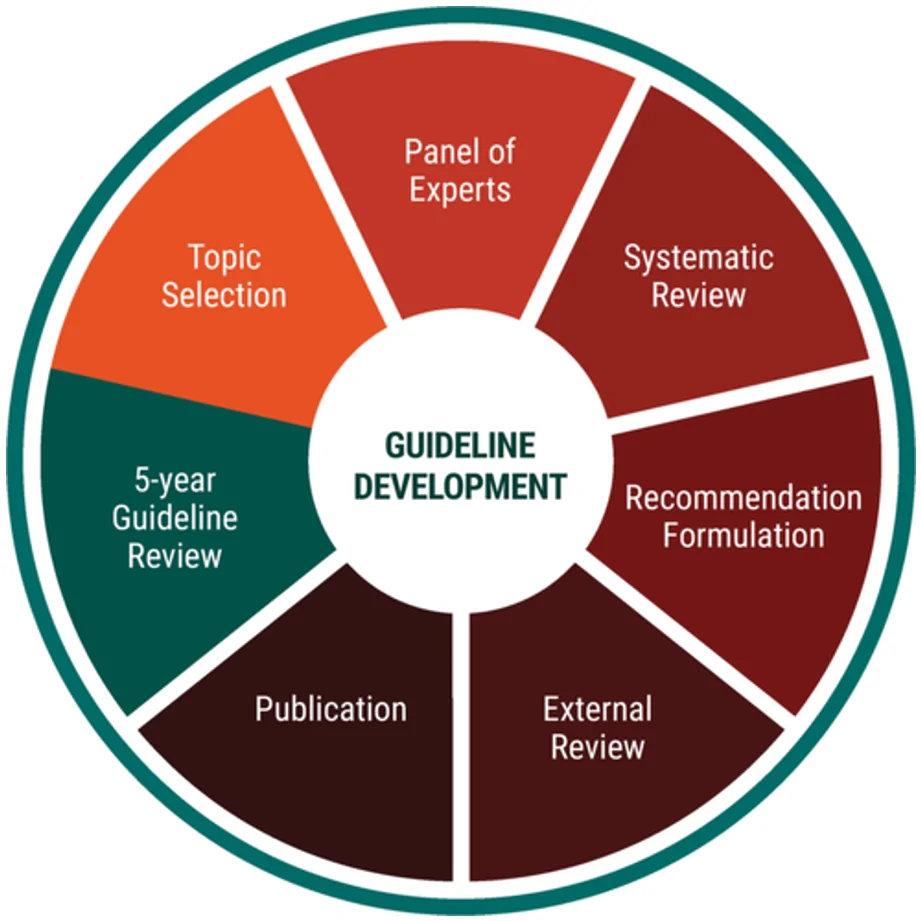
RNAO's guideline development process.

The development process follows seven steps: selecting a BPG topic and determining its purpose and scope, recruiting an expert panel, conducting systematic reviews, developing recommendations and good practice statements, obtaining feedback from the expert panel and external reviewers, publishing the BPG and issuing the next edition every 5 years (Figure 2) [1]. A detailed description of each guideline's development process is published online as a supplemental document. RNAO is examining its process for updating guidelines and exploring the integration of earlier review mechanisms to identify and respond to critical new evidence.

### 2.1 Topic Selection

BPG topics may be updates to existing guidelines or a new area identified based on priorities from BPSOs, the government, public health agencies or public submissions via an online portal [1]. Once a topic is identified, the purpose and scope are refined through an environmental scan of existing guidelines, discussion groups, key informant interviews and a preliminary literature review.

#### 2.1.1 Environmental Scan of Existing Guidelines

Two GDMs are assigned to each BPG and conduct an environmental scan of recently published guidelines on the topic by searching databases and websites. Guidelines are reviewed for content, applicability to health provider scope of practice, accessibility and quality. Each GDM individually evaluates guideline quality using the AGREE II tool [6]. AGREE III is currently under development and, once published, is expected to be incorporated into the appraisal process [8]. The results of these assessments inform decisions regarding the potential adaptation or adoption of existing guidelines [9].

#### 2.1.2 Key Informant Interviews and Discussion Groups

The assigned GDMs consult end‐users through key informant interviews and discussion groups to understand priority areas to be addressed in the BPG. Key informants may include health providers and researchers with expertise in the topic area, PWLE and BPSO representatives familiar with previous guideline editions. Inductive qualitative content analysis is used to analyze the collected data.

#### 2.1.3 Preliminary Literature Review

The preliminary literature review provides an overview of current evidence, including interventions, outcomes and practice trends. Literature may also be reviewed to understand what outcomes and interventions matter to PWLE. A health science librarian conducts database searches to capture relevant studies and the two GDMs independently screen studies using predefined inclusion/exclusion criteria. Selected studies undergo data extraction using a customized form, followed by thematic analysis to inform guideline planning.

### 2.2 Identifying and Appointing Expert Panel Members

Each BPG is supported by an expert panel recruited through open calls on RNAO's website and social media, with targeted outreach to ensure diverse representation [1]. The expert panel's responsibilities include shaping the purpose, scope and research questions, contributing to recommendation development, reviewing BPG drafts, implementation tools and evaluation indicators and promoting dissemination of the BPG.

Each BPG is supported by two co‐chairs with relevant clinical, research or lived experience, one of whom is a PhD prepared registered nurse. The expert panel typically consists of 14–17 members, including nurses, other interprofessional team members and PWLE. Diversity in membership of the expert panel is prioritized through broad representation from health sectors, settings, populations and geographical areas. For health equity‐specific BPGs, most expert panel members must have lived experience. Co‐chairs and expert panel members are confirmed following a review of any actual or perceived conflicts of interest.

Engaging PWLE in guideline panels offers valuable insights into the priorities of those affected by recommendations. However, challenges such as recruitment, comprehension of materials and ensuring meaningful participation may arise. To support involvement, RNAO provides plain‐language resources, support for interpreting evidence and regular check‐ins. An internal committee also meets routinely to refine engagement strategies.

#### 2.2.1 Initial Expert Panel Meetings

Three to four expert panel meetings are held to determine the BPG's purpose, scope and research questions that will inform the systematic reviews. The expert panel also confirms the populations and settings the guideline will cover. Guiding principles are also identified; these are philosophies, values and/or standards that health providers are to integrate into practice when implementing recommendations. Meetings are co‐facilitated by the two panel co‐chairs with support from the two GDMs who hold methodological expertise.

During the orientation meeting, the expert panel is introduced to RNAO's BPG programme, the systematic review process and GRADE approach. Roles, responsibilities and timelines are reviewed. The panel is then sent a survey to gather feedback on priority areas to be included in the guideline.

Additional meetings are held to finalize the purpose, scope and research questions. The RNAO GDMs present findings from the preliminary literature review, discussion groups and key informant interviews. The expert panel then identifies four to six research questions that are critical to address by considering where there is variation or uncertainty in practice and/or existing evidence. The expert panel also discusses outcomes important to decision making and emphasis is placed on considering the perspective of PWLE. A follow‐up survey is sent to rank the top 5 outcomes per question.

### 2.3 Conducting Systematic Reviews

Recommendations are drafted based on a systematic review of the literature. If existing high‐quality GRADE guidelines exist that answer the research question, RNAO may use GRADE‐ADOLOPMENT to adopt or adapt recommendations [9]. If not, recommendations are developed by conducting a new systematic review and the protocol is registered in PROSPERO [10]. In cases where PROSPERO registration is not feasible due to delays or restrictions, alternative registries such as the Open Science Framework (OSF) may be used to ensure transparency [11].

Systematic review questions are developed in accordance with the population, intervention, comparison and outcomes format [12]. For each question, a health sciences librarian conducts searches in key databases, including (but not limited to): Cumulative Index to Nursing and Allied Health (CINAHL), Medline, Medline in Process, Cochrane Central, Cochrane Database of Systematic Reviews, Embase and Emcare. The two GDMs independently screen studies using predefined criteria, resolving disagreements by consensus or a third reviewer. Risk of bias is assessed using validated tools appropriate to each study design: the Cochrane Risk of Bias 2.0 tool, [13] the ROBINS‐I tool, [14] and the ROBIS tool [15]. The two GDMs reach a consensus through discussion. For each study, data are extracted by one GDM and a second GDM completes quality assurance. RNAO is also beginning to integrate the Implementation in Context framework [16] into the data extraction phase as a method of charting contextual details that may support implementation of recommendations.

A qualitative evidence synthesis may also be conducted to obtain insight into the values people place on the outcomes of interest, or the acceptability and feasibility of implementing interventions.

### 2.4 Recommendation Formulation

#### 2.4.1 Determining Certainty of Evidence and Drafting Recommendations

The certainty of quantitative evidence is determined for each prioritized outcome per recommendation [5]. As per GRADE, the certainty of the body of evidence is rated accordingly after assessing for risk of bias, inconsistency, imprecision, indirectness and publication bias [5]. When conflicting evidence is identified, inconsistency is assessed by examining potential sources of heterogeneity in study populations, interventions and outcomes. If the variability remains unexplained, the certainty of evidence may be rated down [17]. An overall certainty of evidence per recommendation is assigned based on assessments and the evidence is deemed to be of high, moderate, low or very low certainty [5]. If a qualitative evidence synthesis is conducted, the confidence in the findings is determined using GRADE‐CERQual [18].

GDMs analyze included studies pertaining to each question and draft recommendations accordingly. For each recommendation, GRADE evidence profiles (EP) are constructed to present decisions on determining the certainty of evidence, and to present general information about the body of evidence, including key statistical or narrative results [5]. In addition, Evidence‐to‐Decision (EtD) frameworks are developed to summarize quantitative and qualitative evidence for formulating recommendation statements [5]. Each EtD document summarizes information about the certainty of evidence, benefits; and harms, values, acceptability, feasibility and health equity implications associated with an intervention.

Expert panel members are provided with the EPs and EtD frameworks to review prior to the recommendation build meetings to determine the direction (i.e., a recommendation for or against use of the intervention) and the strength (i.e., strong or conditional) of the recommendations. Any conflicting evidence is discussed in detail with the expert panel. The strength is suggested by the RNAO GDMs based on the certainty of evidence and finalized by the panel through consensus discussion, facilitated by the co‐chairs.

#### 2.4.2 Good Practice Statements

An additional method of providing guidance in the BPG is through good practice statements. Good practice statements are actionable statements of what should be done in practice [19]. Given the high level of certainty that the benefits derived from the action outweigh the harms, good practice statements are not based on a systematic review of the evidence and they do not receive a rating of the certainty in their evidence or a strength [19, 20]. Good practice statements are interpreted as strong recommendations and are determined following Dewidar et al.'s criteria [19].

#### 2.4.3 BPG Drafting

GDMs draft the BPG once recommendation build meetings are completed following a standard template and style guide. BPGs include background content, guiding principles, recommendations and good practice statements and appendices.

A discussion of evidence accompanies each recommendation. Each discussion of evidence provides an overview of: (1) benefits and harms, (2) values placed on outcomes, (3) acceptability and feasibility of the intervention, (4) health equity considerations and (5) the expert panel's justification of the strength of the recommendation [5]. A discussion and rationale follow the good practice statements supported by peer‐reviewed and grey literature. Implementation tips, derived from evidence and/or the expert panel, are provided to support BPSOs and other end‐users successfully implement the recommendations and good practice statements.

Additional details are provided through supporting resources and appendices that are useful for implementation of the recommendations (e.g., sample tools, images/diagrams, documentation templates). All potential appendices and supporting resources are reviewed independently by two GDMs based on the following criteria: relevance, timeliness, credibility, quality, accessibility and engagement with PWLE in development of the resource. GDMs reach consensus through discussion on each resource prior to inclusion in the BPG.

Concurrent to BPG development, BPG‐specific indicators are also developed and published in monitoring and evaluation charts within the BPG. Expert panel members contribute to the development of structure, process and outcomes indicators according to the Donabedian model [21].

### 2.5 External Review

The draft BPG undergoes several rounds of internal and external review prior to publication. The draft is reviewed by the expert panel and co‐chairs, and RNAO's BPG team including RNAO's CEO. The RNAO BPG team is responsible for ensuring that the guideline is produced in accordance with the RNAO BPG development handbook, the protocol in PROSPERO, GRADE methods and international guideline standards such as AGREE II and the RIGHT statement [5, 6, 7].

RNAO is committed to obtaining feedback on the BPG draft from individuals with subject matter expertise in the guideline topic and those who may be affected by its implementation, including BPSOs. Reviewers include nurses and other health providers, administrators, researchers, educators, nursing students and PWLE. External reviewers for RNAO BPGs are identified through a public call issued on the RNAO website. Individuals and organizations with expertise in the topic area are invited to fill any gaps in representation.

### 2.6 Publication and Dissemination

The final BPG document is published on RNAO's website and made freely available [22]. Supplementary documents are published along with the BPG that detail the systematic review and GRADE process along with declarations of competing interests from panel members. Implementation resources may also be created, including fact sheets and translated versions of the BPG. The BPG is disseminated through numerous channels: website, social media, government officials, RNAO's over 55,000 members, 1500+ national and international BPSOs who systematically implement and evaluate RNAO BPGs, conferences and a webinar outlining the BPG. RNAO publishes at least one manuscript accompanying each BPG.

### 2.7 Five‐Year Guideline Review

RNAO commits to rigorously reviewing each guideline every 5 years after it is published and issuing its next edition.

## 3 Discussion

Guidelines aim to improve the quality of healthcare and reduce variation in care, but specific strategies are needed to help translate evidence from guidelines into clinical practice [23, 24, 25]. The AGREE‐II Instrument contains six domains for assessing the quality of guidelines, one of which is the domain of applicability that involves strategies to promote the uptake, implementation, monitoring and evaluation of guidelines [6]. Unfortunately, clinical practice guidelines frequently fail to sufficiently address applicability [24, 26], and the influence of guideline implementation on clinical outcomes has largely been unquantified [23]. Authors have highlighted the importance of developing robust approaches to evaluate the impact of guideline implementation in practice [23, 27].

RNAO's integrated BPG model was designed to ensure that guidelines are trustworthy and applicable, and that individuals, organizations and health systems are supported to systematically implement guidelines and monitor and evaluate their impact (Figure 1) [1]. When assessed by independent authors using AGREE‐II, RNAO's guidelines have scored high in the domain of applicability [27, 28, 29]. In addition to developing guidelines that include strategies and resources to support implementation and evaluation, RNAO has devoted equal efforts to coach individuals, organizations and health systems implementing guidelines and evaluating their impact. Few guideline development organizations have models that allow them to directly engage with end users and collect qualitative and quantitative feedback about guideline implementation and evaluation in a systematic way. The second and third pillars of RNAO's integrated model, focused on BPG implementation and evaluation, are further described below.

### 3.1 BPG Dissemination, Implementation and Sustainability

The implementation of best practices must be an active, systematic and engaged process, and RNAO supports the uptake and sustainability of BPGs through a multipronged approach that includes individual, organization and system‐level strategies. At the individual level, RNAO has a network of best practice champions. Champions are change agents who are passionate about implementing evidence‐based practices and improving care and health [1]. At the organization level, there are over 1500 BPSOs internationally from over 13 different countries that have partnered with RNAO to systematically implement and evaluate RNAO's BPGs and create evidence‐based practice cultures. At the system level, RNAO Clinical Pathways™ [30] are advancing evidence‐based practices across the long‐term care sector in Ontario by facilitating the integration of BPGs into electronic medical record systems to ensure knowledge translation and optimal health outcomes.

Implementing guidelines at the point of care is multi‐faceted and challenging. It takes more than awareness and access to BPGs for practice to change; guidelines must be adapted for each practice setting in a systematic and participatory way to ensure that recommendations fit the local context [31]. The Leading Change Toolkit [32] provides evidence‐informed processes for guiding uptake and sustainability of BPGs; using two complementary frameworks: the social movement action framework [33, 34] and the knowledge‐to‐action framework [35].

### 3.2 BPG Monitoring and Evaluation

Systematic and ongoing evaluation is pivotal to ethical and effective practice and RNAO supports BPSOs to monitor the uptake and evaluate the impact of BPGs on patient and organizational outcomes [1]. RNAO houses two data systems to support monitoring and evaluation: MyBPSO and Nursing Quality Indicators for Reporting and Evaluation® (NQuIRE®). MyBPSO collects qualitative data about BPG implementation while NQuIRE is an international data system that allows BPSOs to quantitatively measure the impact of BPG implementation. NQuIRE collects, compares and reports data on human resource structure indicators as well as guideline‐specific, nursing‐sensitive structure, process and outcome indicators [24, 36]. This provides a unique opportunity for BPSOs to monitor implementation progress and sustainability through rigorous evaluation. RNAO is currently exploring how real‐world evidence generated through NQuIRE and MyBPSO data can inform the development of next edition BPGs.

### 3.3 Limitations

RNAO strives to rigorously develop guidelines that promote positive outcomes and meet the needs of BPSOs and other end users, but keeping RNAO's library of over 50 BPGs up‐to‐date and balancing new topic requests can be challenging when planning timelines, priorities and resources. In addition, it is feasible for the RNAO guideline development team to conduct four to six systematic reviews accompanying each BPG topic. As such, the breadth of the BPG may be limited and other strategies such as guiding principles or appendices may be used to address content in other areas. Finally, RNAO has encountered challenges using the GRADE approach when developing guidelines that do not have a purely clinical focus, such as those currently being developed with a health equity lens. Enhancing the GRADE approach to better accommodate the unique considerations of health equity‐focused guidelines is an ongoing and future priority.

## 4 Conclusion

RNAO BPGs are developed following a rigorous scoping phase, the GRADE approach and guidance from a diverse expert panel including PWLE. In addition, RNAO places a strong emphasis on equipping GDMs with the competencies to stay up to date with guideline development methodology. RNAO is continually striving to ensure methodological rigour of BPGs that are valuable and applicable to BPSOs and end users. Situating BPG development within an integrated three‐pillar programme that includes guideline dissemination, implementation and sustainability as well as monitoring and evaluation, makes the development process uniquely positioned to improve individual, organizational and health system outcomes.

## Author Contributions

Conceptualization, Lyndsay Howitt, Amy Burt, Christine Buchanan and Nafsin Nizum; funding acquisition, Doris Grinspun; methodology, Nafsin Nizum, Michelle Rey and Doris Grinspun; project administration, Lyndsay Howitt and Amy Burt; writing – original draft, Lyndsay Howitt, Amy Burt and Christine Buchanan; writing – review and editing, Lyndsay Howitt, Amy Burt, Christine Buchanan, Nafsin Nizum, Michelle Rey and Doris Grinspun. All authors have read and agreed to the published version of the manuscript.

## Conflicts of Interest

The authors declare no conflicts of interest.

## Data Availability

Data sharing is not applicable to this article as no data sets were generated or analyzed during the current study.
